# Increased default mode network activity in socially anxious individuals during reward processing

**DOI:** 10.1186/2045-5380-4-7

**Published:** 2014-07-23

**Authors:** Erin L Maresh, Joseph P Allen, James A Coan

**Affiliations:** 1 Department of Psychology, University of Virginia, 314 Gilmer Hall, P.O. Box 400400, Charlottesville, VA 22904, USA

**Keywords:** Social anxiety, Default mode network, Reward, Punishment, Anticipation, Consumption, fMRI

## Abstract

**Background:**

Social anxiety has been associated with potentiated negative affect and, more recently, with diminished positive affect. It is unclear how these alterations in negative and positive affect are represented neurally in socially anxious individuals and, further, whether they generalize to non-social stimuli. To explore this, we used a monetary incentive paradigm to explore the association between social anxiety and both the anticipation and consumption of non-social incentives. Eighty-four individuals from a longitudinal community sample underwent functional magnetic resonance imaging (fMRI) while participating in a monetary incentive delay (MID) task. The MID task consisted of alternating cues indicating the potential to win or prevent losing varying amounts of money based on the speed of the participant’s response. We examined whether self-reported levels of social anxiety, averaged across approximately 7 years of data, moderated brain activity when contrasting gain or loss cues with neutral cues during the anticipation and outcome phases of incentive processing. Whole brain analyses and analyses restricted to the ventral striatum for the anticipation phase and the medial prefrontal cortex for the outcome phase were conducted.

**Results:**

Social anxiety did not associate with differences in hit rates or reaction times when responding to cues. Further, socially anxious individuals did not exhibit decreased ventral striatum activity during anticipation of gains or decreased MPFC activity during the outcome of gain trials, contrary to expectations based on literature indicating blunted positive affect in social anxiety. Instead, social anxiety showed positive associations with extensive regions implicated in default mode network activity (for example, precuneus, posterior cingulate cortex, and parietal lobe) during anticipation and receipt of monetary gain. Social anxiety was further linked with decreased activity in the ventral striatum during anticipation of monetary loss.

**Conclusions:**

Socially anxious individuals may increase default mode network activity during reward processing, suggesting high self-focused attention even in relation to potentially rewarding stimuli lacking explicit social connotations. Additionally, social anxiety may relate to decreased ventral striatum reactivity when anticipating potential losses.

## Background

Although heterogeneous in presentation, anxiety disorders are in part classified based on the shared dimension of heightened negative affect. A stable, higher-order temperamental factor, negative affect encompasses a broad range of distressing emotions, including guilt, hostility, self-dissatisfaction, nervousness, and uneasiness [1,2], that motivate individuals to avoid punishing, threatening, or unfamiliar stimuli [3-5]. This manifests in risk-averse behaviors such as withdrawal and inhibition of behavioral impulses, particularly in the presence of approach/avoidance conflicts [5,6]. Accordingly, high trait levels of negative affect are related to avoidance of both social and non-social threats and may represent a general risk factor for the development and maintenance of anxiety disorders [7-9].

By contrast, there is relatively little work on the role of positive affect in anxiety disorders. Positive affect maintains rewarding, goal-directed behavior, which underlies approach-oriented, appetitive motivation [5,10]. Theoretical and empirical data suggest that anxiety is defined by higher levels of negative affect and withdrawal-related behaviors, while experiences of positive emotions and goal-directed behavior are generally unaffected [4,5,11-13]. Indeed, low positive, approach-related affect is posited to be a feature distinctive to depression [14-17]. Blunted positive emotionality presents as reduced reward-seeking behavior, as well as reduced appetitive and consummatory responses to rewarding stimuli, which characterizes the anhedonia that frequently accompanies depression [18,19].

Accumulating research suggests that social anxiety disorder is an exception to the view that positive affect and corresponding approach-oriented behavior are not altered in anxiety. In assessing associations between positive and negative affect in depression and anxiety diagnoses among 350 outpatients, Brown et al. [20] found low positive affect to associate solely with social anxiety disorder among the anxiety disorders and, further, to be approximately equally related to social anxiety disorder and depression. More recent research has examined how socially anxious individuals experience altered positive affect in daily life. Individuals with high social anxiety experience positive emotions and events both less intensely and less frequently than those with less social anxiety, particularly on days when they are feeling especially socially anxious [21,22]. It has been proposed that high levels of behavioral inhibition and low levels of approach motivation act as both distal and proximal causes for the development and maintenance of social anxiety [23,24].

Although altered experiences of positive and negative affect in social anxiety have primarily been observed in self-report and behavioral studies, less is known about how they may be represented on the neural level. Some research suggests altered striatal morphology and function in individuals with social anxiety disorder [25], yet how this interferes with behavior, particularly during situations not explicitly social in nature, is poorly understood. One way to address these issues is to examine how social anxiety interacts with neural responses during incentive processing using tasks developed for use in the functional magnetic resonance imaging (fMRI) environment. The monetary incentive delay (MID) task [26] uses visual cues to indicate the potential of winning or losing varying amounts of money based on speed in response to a subsequent target cue. This task allows for analysis of multiple phases of incentive processing, including the anticipation phase and the outcome phase. The MID task produces robust activity in basal ganglia during anticipation of incentives and in the orbital and medial prefrontal cortices (MPFC) during consumption of incentives [27-29].

Using the MID task, we sought to identify whether levels of social anxiety moderated the neural response to both the anticipation and consumption of reward and punishment. Due to reported differences in the experience of positive and negative affective states between social anxiety and other types of anxiety, we were additionally interested in comparing effects due to social anxiety with effects due to generalized trait anxiety on neural reward and punishment anticipation. To this end, we examined whether continuous levels of social and trait anxiety in a large, non-clinical community sample would be predictive of differences in reward and punishment sensitivity.

Because social anxiety is related to altered positive and negative affect, we expected to find associations between social anxiety and neural activity in response to processing monetary incentives. Specifically, we hypothesized that: (1) during the anticipation of monetary incentives, higher levels of social anxiety would associate with decreased activity in reward-related regions such as the ventral striatum, and (2) during monetary incentive outcomes, higher levels of social anxiety would associate with decreased activity in regions previously found to be related to reward consumption, such as the MPFC. These hypotheses were explored using both whole brain and region of interest (ROI) analyses targeted on the ventral striatum for the anticipation phase and the medial prefrontal cortex for the outcome phase.

## Methods

### Participants and questionnaire data

Participants from a larger longitudinal study on adolescent social development [30,31] were invited to participate in an fMRI study. This sample has been followed for over 12 years, beginning when participants were young teenagers. For the present study, participants were excluded if they were pregnant or exhibited risk for incident in the fMRI environment. The final sample consisted of 84 participants (42 women) who predominantly self-identified as Caucasian (n = 45) or African American (n = 33). Average age of participants at the time of the scan was 24.56 (SD = 1.17) years old. No participants reported having clinical social anxiety disorder, although two reported having bipolar disorder via self-report on the Medical Information Questionnaire. Excluding these two participants did not alter the results, so they are included in all analyses. No participants reported taking any psychiatric medications at the time of the study. All participants gave written consent and were provided monetary compensation for their time. The study was approved by the University of Virginia Institutional Review Board for Health Sciences Research (#12984).

During each wave of data collection, participants completed a battery of questionnaires on personality, attachment style, and relationships. Because the longitudinal study began when participants were adolescents, levels of social anxiety were measured using the Social Anxiety Scale for Adolescents (SAS-A) [32]. The SAS-A is a self-report questionnaire consisting of 22 questions (18 social anxiety-related questions and four filler questions) that participants answer using a five-point Likert scale to indicate how much the statement describes them. The SAS-A generally loads onto three factors: Fear of Negative Evaluation (FNE) (eight items, for example, ‘I worry about what others think of me’), Social Avoidance and Distress in New Situations (SAD-New) (six items, for example, ‘I get nervous when I meet new people’), and Social Avoidance and Distress in General Situations (SAD-General) (four items, for example, ‘I feel shy even with people I know well’) [33]. For most of our analyses, we used a total social anxiety score determined by summing these three subscales, resulting in a possible range of scores from 18 to 90. We also examined associations between individual subscales and neural activity. To assess levels of trait anxiety, we used scores on the Trait portion of the State-Trait Anxiety Inventory (STAI) [34], which has a possible range of scores from 20 to 80. Each scale, the SAS-A and STAI, had seven waves of data available in our longitudinal sample, dating from when participants were an average of 18.24 (SD = 1.05) years old to an average of 25.28 (SD = 0.88) years old. This last wave of data was collected an average of 295.30 (SD = 250.18) days after the fMRI scan. The SAS-A and STAI scales showed excellent test-retest reliability (SAS-A: Cronbach’s alpha = 0.92; STAI: Cronbach’s alpha = 0.90). Thus, we collapsed across the available waves for each participant, yielding an average SAS-A and STAI score across time, indicative of a stable trait for each participant.

### Monetary incentive delay task

**Figure 1:**
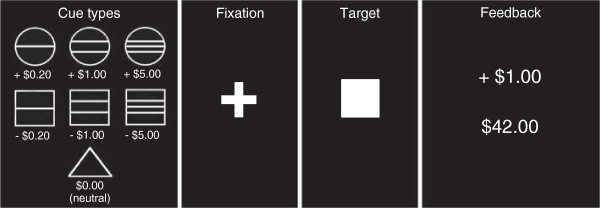
**Monetary incentive delay (MID) task.** Each run of the MID task consisted of 72 trials. The first box shows the cue types presented, with circles indicating the potential to win money (gain cue), squares indicating the potential to lose money (loss cue), and a triangle indicating no money will be won or lost (neutral cue). A cue was presented for 500 ms, followed by a fixation cross (2,000 to 2,500 ms) and then the target square (160 to 260 ms), during which the participant was instructed to press a button as quickly as possible to win or avoid losing money. A feedback screen (1,650 ms), in which the top number indicated the amount of money won or lost during that trial and the bottom number indicated the participant’s total amount, was presented at the end of each trial.

The monetary incentive delay (MID) task is designed to assess one’s neural response to the anticipation and receipt of rewarding or punishing monetary stimuli [27]. Participants entered an fMRI scanner and underwent two runs of the MID task, each consisting of 72 trials for a total of 144 trials (Figure 1). During each trial, one of seven possible cue shapes was presented for 500 ms (anticipation phase). Three of the cue shapes indicated the potential to win varying amounts of money (‘gain’ cues, represented by circles, n = 54), three of the cue shapes indicated a potential to lose varying amounts of money (‘loss’ cues, represented by squares, n = 54), and one cue shape indicated no money would be won or lost (‘neutral’ cue, represented by a triangle, n = 36). Horizontal lines across each shape represented the amount of money one could potentially win or lose, with one line signifying the potential to win or lose $0.20, two lines signifying $1.00, and three lines signifying $5.00. After presentation of the cue, a fixation cross was displayed for 2,000 to 2,500 ms, followed by presentation of a white target square for 160 to 260 ms. Participants were instructed that, upon seeing the target square, they were to press a button on a provided button box as quickly as possible to gain or avoid losing money. A practice trial before the two runs calibrated the length of time the target square was presented so that all participants accurately pressed the button approximately 80% of the time. Trial outcomes were based on actual performance. A feedback screen (outcome phase), presented for 1,650 ms, displayed whether they had won or lost money during that trial and their cumulative earnings. Each participant was initially given $40 and was paid the final amount of money earned based on their performance.

### Image acquisition

Images were acquired using a Siemens 3.0 Tesla MAGNETOM Trio high-speed magnetic imaging device with a circular polarized transmit/receive head coil and integrated mirror. A total of 176 high-resolution T1-magnetization-prepared rapid-acquisition gradient echo images were collected to determine the localization of function (1-mm slices, repetition time = 1,900 ms, echo time = 2.53 ms, flip angle = 9°, field of view = 250 mm, voxel size = 1 × 1 × 1 mm). A total of 224 functional T2*-weighted echo planar images sensitive to blood oxygen level-dependent (BOLD) contrasts were collected per block in volumes of 28 3.5-mm transversal echo-planar slices covering the whole brain (1-mm slice gap, repetition time = 2,000 ms, echo time = 40 ms, flip angle = 90°, field of view = 192 mm, matrix = 64 × 64, voxel size = 3 × 3 × 3.5 mm).

Data were preprocessed using FMRIB Software Library (FSL) software (Version 5.0.5; http://www.fmrib.ox.ac.uk/fsl). Motion was corrected using FMRIB Linear Image Registration Tool (FLIRT), an intra-modal correction algorithm tool [35], with slice scan-time correction and a high-pass filtering cutoff point of 100 s, removing signals that were irrelevant to the stimuli. We used FSL’s Brain Extraction Tool (BET) to eliminate unwanted, non-brain material voxels in the fMRI data. Spatial smoothing was conducted with a 5-mm full width at half minimum Gaussian kernel. Images were registered to the Montreal Neurological Institute (MNI) standard space by FLIRT [35].

### Data analysis

Data analysis was conducted using fMRI Expert Analysis Tool (FEAT) Version 6.00 in the FSL software package. Both the anticipation and outcome phases of incentive processing are captured by the MID task, and similar analyses were conducted for each. At the first level of analysis, reward and punishment maps were created by subtracting the neural response to the neutral anticipation and outcome cues from the neural response to the gain or loss anticipation and outcome cues, respectively. We collapsed across all amounts of gain or loss ($0.20, $1.00, and $5.00) to examine overall neural activity attributable to each. At the second level of analysis, we combined data from the two runs of the MID task for each participant using a fixed effects model. At the third level of analysis, we ran our primary model of interest - a mixed effects model taking into account within-subject fixed effect variance and between-subject random effect variance - for reward and punishment contrasts in both the anticipation phase and the outcome phase, with total SAS-A scores entered as a covariate. This model was used to determine both main effects of gain and loss during the anticipation and outcome phases, as well as to determine regions differentially associated with social anxiety.

We conducted several complementary models for both gain and loss in the anticipation and outcome phases to examine nuances in the role of social anxiety in reward and punishment processing and to rule out alternative explanations. For example, to assess the degree to which social anxiety uniquely impacts incentive processing, we conducted a model in which both social anxiety and trait anxiety scores were entered as covariates. Detailed results of the following analyses can be found in the Additional file 1. First, to explore differences in neural reactivity based on incentive magnitude, we examined contrasts of large ($5.00) minus small ($0.20) incentives for gain and loss in the anticipation and outcome phases. Next, to determine which specific facets of the SAS-A relate to brain activity, we conducted separate models for each of the three individual subscale scores (FNE, SAD-New, and SAD-General). Following that, we sought to examine the degree to which trait anxiety corresponds with brain activity in regions implicated in our analyses. This was accomplished by assessing the impact - if any - of trait anxiety within clusters we found to co-vary with social anxiety scores. Finally, we explored differences related to social anxiety in gain *versus* loss contrasts for the anticipation and outcome phases. Because of previous findings suggesting gender differences in incentive processing [36], we compared analyses with gender entered as a covariate to those without. Including gender as a covariate yielded highly similar results; therefore, all analyses reported do not include gender.

To correct for multiple comparisons within models, we performed a whole brain, cluster-wise correction derived from Gaussian Random Field (GRF) theory (see [37]). To balance risk of type I and type II errors [38], a GRF-corrected maximum height threshold of z >1.96 was used to define contiguous clusters [39]. Cluster significance levels were then compared with a family-wise error (FWE) corrected cluster-significance threshold of *P* <0.05. For each cluster, we report the location of the peak voxel as well as five local maxima (voxels with surrounding voxels of lower intensities). Anatomical labels for cluster maxima were identified using the Harvard-Oxford cortical and subcortical structural atlases or the Juelich histological atlas if no label was available in the Harvard-Oxford atlases. All coordinates are reported in MNI space.

Because our initial hypotheses involved reward-related regions, we additionally performed region-of-interest (ROI) analyses using a mask of the bilateral ventral striatum for the anticipation phase and a mask of the MPFC for the outcome phase, both thresholded at an uncorrected *P* value of *P* <0.005 with total social anxiety scores entered as a covariate. These masks were derived from the nucleus accumbens structure and the frontal medial cortex structure in the Harvard-Oxford subcortical structural atlas. To further describe interactions between social anxiety and ventral striatum or MPFC activity, we examined complementary models for both the anticipation and outcome phases: one with both social and trait anxiety scores entered as covariates and three including each of the social anxiety subscales. Clusters were defined as areas of at least 10 contiguous significantly active voxels.

## Results

### Questionnaire results

Multiple waves of questionnaire data for social and trait anxiety scores were available for all 84 participants, with the number of available waves for each participant in the range of 3 to 7 (*M* = 6.30, SD = 0.95). Average total social anxiety scores on the SAS-A ranged from 18.43 to 63.14 (*M* = 31.18, SD = 9.49), and average trait anxiety scores from the STAI ranged from 22.33 to 56.86 (*M* = 35.37, SD = 7.48). Both SAS-A scores and STAI scores were slightly positively skewed (SAS-A skewness = 0.81, SE = 0.26; STAI skewness = 0.44, SE = 0.26). However, log-transforming the variables did not substantially change the results; therefore, untransformed data were used in all analyses. Social anxiety and trait anxiety scores were moderately correlated (*r* = 0.58, *P* <0.0001). Social anxiety scores did not differ by gender (*P* = 0.47); however, trait anxiety scores showed a trend toward differing by gender (*P* = 0.06). Follow-up testing showed that women trended toward reporting higher trait anxiety scores (*M* = 36.90, SD = 7.70) than men (*M* = 33.83, SD = 7.01).

### Behavioral data

Reaction times (RTs) significantly differed between gain and loss cue types, *F*(1, 82) = 16.32, *P* < 0.0005, η_p_^2^ = 0.16, such that participants were significantly faster reacting to gain cues (*M* = 0.270 s, SD = 0.04 s) than to loss cues (*M* = 0.278 s, SD = 0.04 s). Neither social anxiety nor trait anxiety had a significant effect on RTs by cue type, *F*(1, 82) = 3.27, *P* = 0.07, η_p_^2^ = 0.04 and *F*(1, 82) = 0.56, *P* = 0.46, η_p_^2^ = 0.01, respectively, although the nearly significant association between social anxiety and RT warranted a closer look. Follow-up analysis revealed that individuals higher in social anxiety trended toward greater reaction times to gain cues but not to loss cues. Neither social anxiety nor trait anxiety showed a significant effect on hit rate, *F*(1, 82) = 0.34, *P* = 0.56, η_p_^2^ = 0.004, and *F*(1, 82) = 0.01, *P* = 0.93, η_p_^2^ < 0.0005, respectively.

### fMRI data

For quick reference, a summary of significant findings can be found in Table 1.

**Table 1:** Significant associations between social anxiety and brain activity in whole brain and ROI analyses

	**Anticipation**		**Outcome**	
**Whole brain**	**Gain**	**Loss**	**Gain**	**Loss**
Total SA	+		+	
FNE	+		+	+
SAD-New	+		+	
SAD-Gen				-
SA with TA	+		+	
**Ventral striatum**				
Total SA		-		
FNE		-		
SAD-New		-		
SAD-Gen	-			
SA with TA		-		
**MPFC**				
Total SA				
FNE				
SAD-New				
SAD-Gen				
SA with TA				

Ventral striatum analyses were conducted for the anticipation phase only; MPFC analyses were conducted for the outcome phase only.

FNE = Fear of Negative Evaluation subscale; SA = social anxiety; SAD-Gen = Social Avoidance and Distress in General Situations subscale; SAD-New = Social Avoidance and Distress in New Situations subscale; TA = trait anxiety; + = positive association with brain activity; − = negative association with brain activity.

## Anticipation phase

### Main effects of gain anticipation

**Figure 2:**
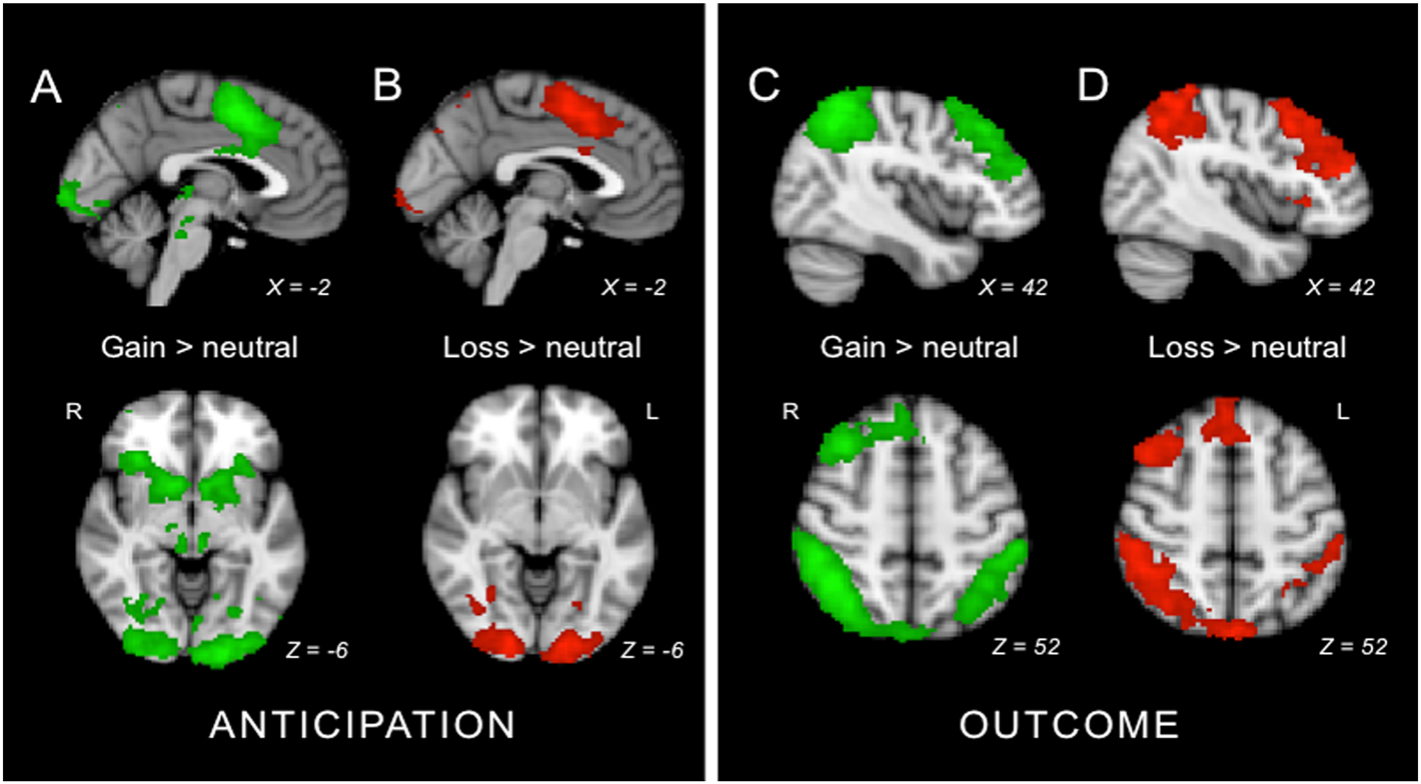
**Main effects of reward and punishment in anticipation and outcome phases.** Colored regions indicate clusters more active during **(A)** anticipation during gain *versus* neutral cues, **(B)** anticipation during loss *versus* neutral cues, **(C)** outcome after gain *versus* neutral cues, and **(D)** outcome after loss *versus* neutral cues. Clusters were identified using whole brain, cluster-wise correction with a z threshold of 1.96 and a corrected cluster significance threshold of *P* <0.05.

Anticipation of monetary reward was analyzed by contrasting neural activity during anticipation of gain cues with neural activity during anticipation of neutral cues. This contrast yielded extensive bilateral activations in regions previously found to be active in response to reward anticipation (for example, Knutson et al., 2001 [27,28]). These regions included the supplementary motor cortex, paracingulate cortex, ACC, superior frontal gyrus, precentral gyrus, postcentral gyrus, right supramarginal gyrus, angular gyrus, superior parietal lobule, precuneus, temporal occipital fusiform cortex, lateral occipital cortex, occipital pole, insula, putamen, caudate, nucleus accumbens, and brainstem (Additional file 2: Table S1, Figure 2A). The reverse contrast, neutral *versus* gain anticipation, is described in Additional file 1 and Additional file 2: Table S1.

### Social anxiety and gain anticipation

**Figure 3:**
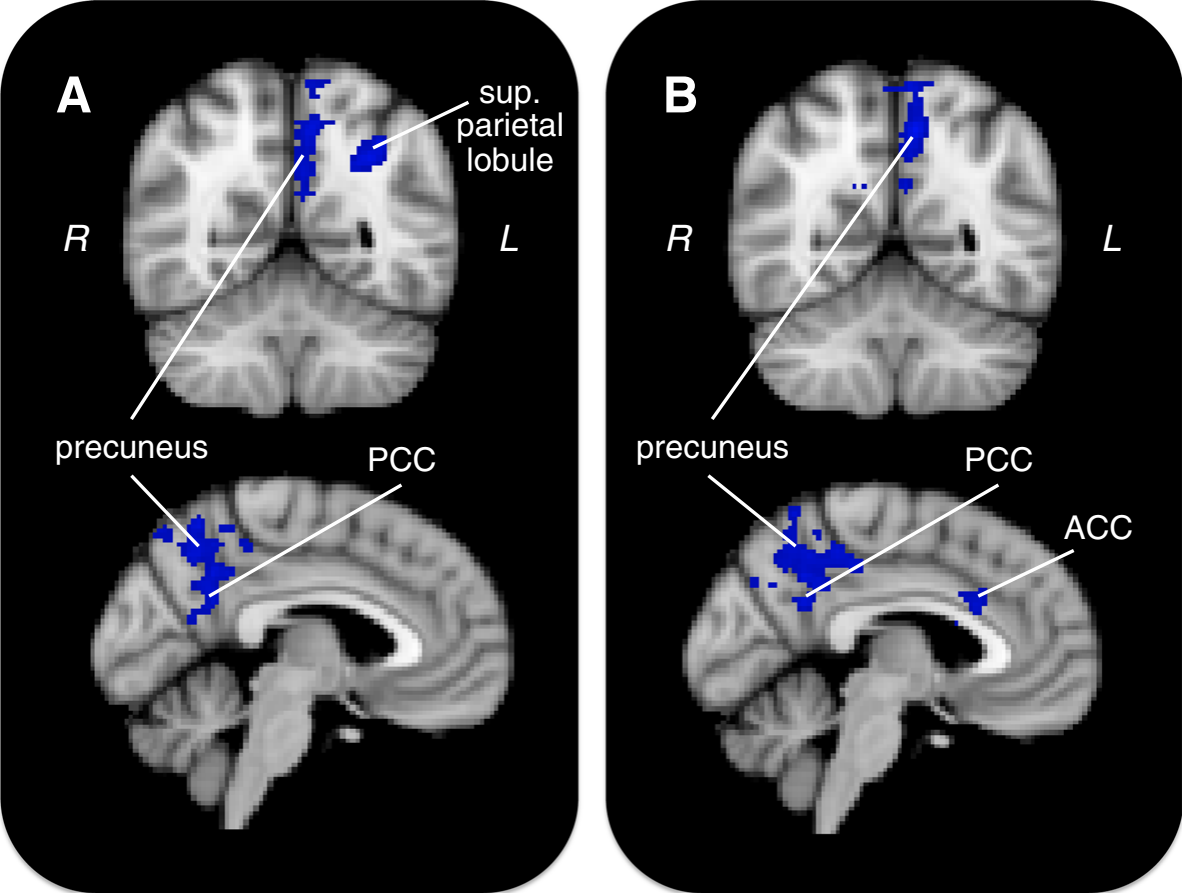
**Clusters related to social anxiety during reward anticipation.** Colored regions indicate clusters positively associated with social anxiety in the gain anticipation > neutral anticipation contrast. Shown are models **(A)** with social anxiety alone, and **(B)** with both social and trait anxiety included. For top row, Y = −56; for bottom row, X = −4. ACC = anterior cingulate cortex; PCC = posterior cingulate cortex.

Our primary research question was whether social anxiety was linked to altered neural reactivity during reward processing. Higher levels of social anxiety predicted increased gain anticipation activity in one main cluster (Figure 3A, Table 2). This cluster peaked in the right medial parietal lobe and extended to the right precuneus, posterior cingulate cortex, angular gyrus, superior parietal lobule, supramarginal gyrus, and lateral occipital cortex. Social anxiety was more strongly related to activity during anticipation of small compared to large rewards (Additional file 1 and Additional file 2: Table S2). Further, brain activity specifically associated with scores on the FNE and SAD-New subscales (Additional file 1 and Additional file 2: Table S3).

**Table 2:** Positive associations with social anxiety in gain > neutral anticipation contrast using whole brain analysis

**Cluster *P *value**	**Cluster size**	**Peak Z**	**x**	**y**	**z**	**Region**	**R/L**
**Model with SA only**							
<0.01	966	3.21	−18	−40	36	Callosal body	L
		3.12	−30	−56	36	Intra-parietal sulcus	L
		3.12	−8	−54	48	Precuneus	L
		3.08	0	−78	52	Precuneus	R/L
		2.98	−34	−56	42	Superior parietal lobule	L
		2.97	−6	−54	36	Precuneus	L
**Model with SA + TA**							
<0.001	1,258	3.57	−20	44	18	Frontal pole	L
		3.56	−26	34	42	Middle frontal gyrus	L
		3.44	−20	42	10	Callosal body	L
		3.23	−28	56	12	Frontal pole	L
		3.15	−22	34	28	Middle frontal gyrus	L
		3.15	−22	16	36	Unclassified white matter	L
<0.01	1,085	3.48	−8	−56	48	Precuneus	L
		3.25	−10	−66	34	Precuneus	L
		3.15	−8	−42	48	Precuneus	L
		3.09	−6	−54	38	Precuneus	L
		2.89	−6	−50	34	Posterior cingulate cortex	L
		2.89	−10	−52	38	Precuneus	L

L = left hemisphere; R = right hemisphere; SA = social anxiety; TA = trait anxiety.

#### With trait anxiety

Due to conceptual overlap between social anxiety and trait anxiety, we investigated whether social anxiety made unique contributions to predicting neural responses to reward anticipation beyond that provided by trait anxiety. When including trait anxiety scores in the model, social anxiety remained predictive, associating with increased activity in two clusters (Figure 3B, Table 3). The first cluster peaked in the left frontal pole and extended to the left middle frontal gyrus and ACC. The second cluster peaked in the left precuneus and extended to the left PCC.

**Table 3:** Positive associations with social anxiety in gain > neutral outcome contrast using whole brain analysis

**Cluster *P *value**	**Cluster size**	**Peak Z**	**x**	**Y**	**z**	**Region**	**R/L**
**Model with SA only**							
<0.0001	2,152	3.82	−36	−42	54	Superior parietal lobule	L
		3.78	−52	−32	50	Supramarginal gyrus	L
		3.59	8	−48	60	Precuneus	R
		3.54	−36	−54	54	Superior parietal lobule	L
		3.50	−42	−48	56	Superior parietal lobule	L
		3.49	−10	−42	54	Postcentral gyrus	L
**Model with SA + TA**							
<0.0001	1,392	3.43	−54	−50	46	Supramarginal gyrus	L
		3.41	−54	−34	50	Supramarginal gyrus	L
		3.30	−36	−44	54	Superior parietal lobule	L
		3.27	−42	−48	56	Superior parietal lobule	L
		3.26	−46	−50	56	Supramarginal gyrus	L
		3.23	−50	−50	50	Supramarginal gyrus	L

L = left hemisphere; R = right hemisphere; SA = social anxiety; TA = trait anxiety.

#### Ventral striatum ROI analysis

To directly assess our hypothesis that social anxiety would correspond with decreased activity in reward-related regions during reward processing, we used an ROI of the bilateral ventral striatum. Social anxiety, whether by itself or with trait anxiety included in the model, showed no association with activity in this region during anticipation of gain. However, when looking at subscales of the SAS-A, right ventral striatum activity showed a negative association with scores on the SAD-General subscale.

### Main effects of loss anticipation

Anticipation of monetary loss was analyzed by contrasting neural activity during loss anticipation cues with neural activity during neutral anticipation cues. This contrast resulted in bilateral activations similar to those seen in anticipation during gain (Figure 2B, Additional file 2: Table S4). These activations were found in regions including the lateral occipital cortex, occipital pole, occipital fusiform gyrus, temporal occipital fusiform gyrus, superior parietal cortex, supramarginal gyrus, angular gyrus, middle frontal gyrus, superior frontal gyrus, precentral gyrus, paracingulate, ACC, and supplementary motor cortex. The reverse contrast, neutral *versus* loss anticipation, is described in Additional file 1 and Additional file 2: Table S4.

### Social anxiety and loss anticipation

Unlike anticipation during gain cues, anticipation during loss cues compared to anticipation during neutral cues did not reveal any clusters of activity significantly related to social anxiety. This remained after including trait anxiety scores in the model.

#### Ventral striatum ROI analysis

During anticipation of loss, a cluster in the right ventral striatum emerged as negatively related to social anxiety. This result remained after including trait anxiety in the model. In particular, ventral striatum activity was negatively related with scores on the FNE and SAD-New subscales.

### Gain *versus* loss anticipation with social anxiety

To further explore the role of social anxiety in gain and loss anticipation, we examined gain *versus* loss anticipation with social anxiety entered as a covariate. Social anxiety was not significantly associated with neural activity in this contrast. This is not altogether surprising due to the similarity in brain activity during gain and loss anticipation trials. In other words, while there is a large difference between gain/loss anticipation and neutral anticipation, the variability between gain and loss anticipation may be too small to observe an effect associated with social anxiety.

## Outcome phase

### Main effects of gain outcome

Monetary reward outcome was analyzed by contrasting neural activity during gain outcome cues with neural activity during neutral outcome cues. Contrasting gain outcome cues with neutral outcome cues yielded activation in predictable regions including the bilateral angular gyrus, supramarginal gyrus, superior parietal lobule, precuneus, and lateral occipital cortex, and right middle and superior frontal gyri, dorsolateral prefrontal cortex, and paracingulate (Additional file 2: Table S5, Figure 2C). The reverse contrast, neutral *versus* gain outcome, is described in Additional file 1 and Additional file 2: Table S5.

### Social anxiety and gain outcome

**Figure 4:**
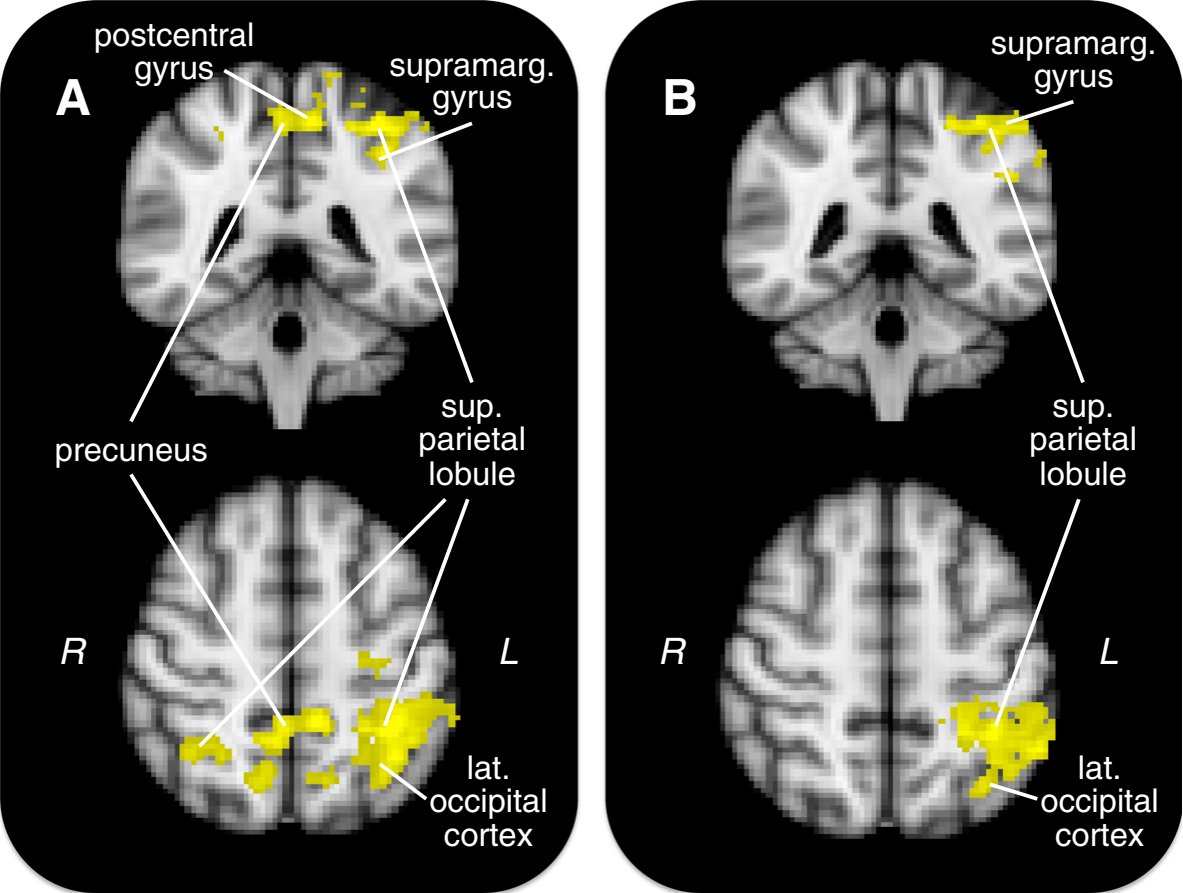
**Clusters related to social anxiety during reward outcome.** Colored regions indicate clusters positively associated with social anxiety in the gain outcome > neutral outcome contrast. Shown are models **(A)** with social anxiety alone, and **(B)** with both social and trait anxiety included. For top row, Y = −42; for bottom row, Z = 54.

Contrasting gain outcome with neutral outcome yielded one cluster positively associated with social anxiety (Table 3, Figure 4A). This cluster peaked in the left superior parietal lobule and extended to bilateral lateral occipital cortex and right superior parietal lobule, precuneus, left supramarginal gyrus, precentral gyrus, and postcentral gyrus. Social anxiety associated with greater activity in large gain outcomes compared to small gain outcomes (Additional file 1 and Additional file 2: Table S6). Similarly to gain anticipation, the FNE and SAD-New subscales, but not SAD-General, showed a relationship with brain activity during gain outcome (Additional file 1 and Additional file 2: Table S7).

#### With trait anxiety

With both social and trait anxiety in the same model, social anxiety showed a positive association with activity in a cluster similar to that seen with social anxiety alone, although the cluster was less extensive and was confined to the left hemisphere (Table 3, Figure 4B). This cluster peaked in the left supramarginal gyrus and extended to the left superior parietal lobule, angular gyrus, and postcentral gyrus.

#### MPFC ROI analysis

Using an ROI of the MPFC revealed no associations between social anxiety and brain activity during gain outcome. Subsequent analyses including trait anxiety or examining specific social anxiety subscales also showed no associations.

### Main effects of loss outcome

Contrasting loss outcome cues with neutral outcome cues resulted in activations in areas including bilateral supramarginal gyrus, angular gyrus, superior parietal lobule, superior frontal gyrus, paracingulate, lateral occipital cortex, cuneus, precuneus, and right frontal pole, middle frontal gyrus, inferior frontal gyrus, and insula (Additional file 2: Table S8, Figure 3D). The reverse contrast, neutral *versus* loss, is described in Additional file 1 and Additional file 2: Table S8.

### Social anxiety and loss outcome

Social anxiety, both by itself and with trait anxiety in the model, was not significantly associated with neural activity when contrasting loss outcome cues with neutral outcome cues. However, examining subscales revealed significant relationships: brain activity during gain outcome showed a positive association with the FNE subscale and a negative association with the SAD-General subscale (Additional file 1 and Additional file 2: Table S9).

#### MPFC ROI analysis

Examining loss outcome using an ROI of the MPFC yielded no relationship with social anxiety, whether using total scores, subscale scores, or adjusting for trait anxiety.

### Gain *versus* loss outcomes with social anxiety

Contrasting activity during gain compared to loss outcomes yielded one cluster positively related to social anxiety that peaked in the left supramarginal gyrus and extended to the left angular gyrus, superior parietal lobule, and lateral occipital cortex (Additional file 2: Table S10). In other words, individuals higher in social anxiety showed greater activity in these areas during gain outcomes than during loss outcomes.

## Discussion

Emerging evidence suggests that social anxiety disorder is unique among anxiety disorders in that it is characterized not only by increased experiences of negative affect but also by decreased experiences of positive affect. It is unclear how this may be reflected in the brain and whether this is seen solely in response to social cues. We used a well-known and much replicated fMRI paradigm, the monetary incentive delay (MID) task, to investigate how levels of social anxiety moderate brain activity during both the anticipation and receipt of non-social reward and punishment in a non-clinical community sample.

Our findings add to the growing body of literature showing altered incentive processing in social anxiety and extend previous findings to include non-social reward and punishment; however, our results were not exactly as expected. As predicted, greater social anxiety corresponded with reduced activity in the ventral striatum during anticipation of loss, suggesting reduced anticipatory reward-related processing when potentially avoiding an aversive stimulus. However, contrary to our hypothesis based on work linking social anxiety with reduced positive experience, we found little relationship between ventral striatum activity and social anxiety during gain anticipation, except when specifically examining the SAD-General subscale [17,21,22,25,41]. Interestingly, depression, which is defined in large part by decreased positive affect, also does not co-vary with reduced ventral striatum activity in response to anticipation of reward [42,43]. When examining gain and loss outcomes using an ROI of the MPFC, we found no associations between brain activity and social anxiety. Strikingly, however, whole brain analysis revealed positive associations between social anxiety and regions such as the precuneus, PCC, and parietal lobe - areas implicated in the default mode network (DMN) - during both the anticipation and receipt of reward. These associations held even after partialing out variance due to trait anxiety.

Activity in the DMN, a collection of brain areas usually more active during rest and less active during task-focused activities [40], is thought to reflect, among other things, self-referential processing, mind-wandering, and episodic memory [44]. That these areas were more active during reward processing among individuals high in social anxiety may indicate that socially anxious individuals find it difficult to direct attention away from the self and toward positive stimuli. This may be especially true in novel situations and when one fears evaluation by others, as similar findings were observed for both anticipation and outcome phases when using the FNE and SAD-New subscales of the SAS-A, but not when using the SAD-General subscale. It is important to note that in these areas, participants generally showed decreased activity during gain compared to neutral cues, as would be expected in DMN areas. Thus, the ‘increased’ activity seen in more socially anxious individuals is, more accurately, less of a decrease. Social anxiety may correspond with a smaller shift in processing from self-focused, ‘default’ processing to generalized reward processing. Interestingly, many participants, particularly those in the upper half of social anxiety scores, actually showed greater activity in DMN areas during gain relative to neutral trials.

Precuneus/PCC activity is increased during self-processing and reflection and decreased during goal-directed, non-self-referential activity [45]. The lower reduction in activity in these brain areas seen in the more socially anxious individuals in our sample may indicate a continued need to maintain self-focused vigilance during a state of anticipatory reward, possibly due to assigning greater motivational salience to anticipation of reward compared to the less socially anxious individuals. This pattern of brain activity was also seen in socially anxious individuals during the outcome phase, suggesting self-focused attention may continue even when a socially anxious person’s need to perform is over and a reward is being processed. Indeed, research suggests that individuals with social anxiety show not only a fear of negative evaluation but also a fear of positive evaluation [46], which may be due to socially anxious individuals predicting they will not be able to live up to future standards when they perform well. Because participants in our study maintained high hit rates (around 80%), it is possible that the more socially anxious individuals felt added pressure to continue performing well, which may have been mediated by activity in DMN areas.

Our findings somewhat contrast with a study examining both social and non-social reward processing in people with clinical social anxiety disorder [47]. In that study, individuals with social anxiety disorder (n = 15) showed no neural differences from healthy controls (n = 19) while anticipating and receiving monetary reward during the MID task. However, decreased activity in the accumbens was observed in the socially anxious group when anticipating social reward (in this case, a picture of smiling face). It is possible that our much larger sample size (n = 84) afforded the ability to identify brain regions related to social anxiety during anticipation of monetary reward due to a level of statistical power unavailable in the prior study.

Importantly, other constructs related to social anxiety can alter reward processing during incentive delay tasks. For example, shy individuals exhibit relatively quicker reaction times than non-shy individuals to target stimuli when anticipating rewards compared to punishments or neutral incentives, suggesting increased reward sensitivity in shy people [48]. Similar behavioral differences based on social anxiety levels were not found in our study, however. Instead, our more socially anxious participants trended toward slower reaction times for reward incentives. It is possible that shyness differs from social anxiety in some way relevant to reaction times - perhaps shy people lack the same fear of evaluation that may hinder performance in socially anxious individuals. In other work, adolescents who had been classified as behaviorally inhibited in childhood showed increased striatal activity when anticipating incentives compared to those who had not been classified as behaviorally inhibited [49]. These (and our own) findings run counter to our hypothesis that socially anxious people should show decreased reward sensitivity. Importantly, however, Guyer et al. suggest that the increased striatal activity in behaviorally inhibited adolescents is due to heightened concern about making errors - that behaviorally inhibited participants may have been more vigilant during larger incentives due to fear of failure. Corroborating this was the finding that affective ratings for gain cues given after the experiment did not differ between the two groups (that is, behaviorally inhibited individuals did not rate gain cues more positively than non-behaviorally inhibited individuals), suggesting that increased striatal activity did not correspond with increased self-reports of positive experiences.

In the present study, social anxiety showed fewer and less consistent relationships with brain activity during both loss anticipation and loss outcomes. This seems counter-intuitive, given that fear-related disorders are prominently characterized by anxiety about upcoming negative events. Whether this is due to an actual absence of a relationship between social anxiety and punishment processing, due to our lack of a clinically socially anxious group, or due to the high hit rates in our sample is unclear. Of note, when taking an ROI approach, social anxiety corresponded with decreased activity in the ventral striatum during anticipation of loss. Anticipation of punishment generally does not recruit ventral striatum activity [27], as was the case in our sample. This suggests that the more socially anxious individuals in our sample experienced a decrease in ventral striatum activity during loss anticipation compared to neutral anticipation, rather than just less of an increase. However, loss outcomes (in which participants generally successfully avoided punishment) did not show differential MPFC activity related to social anxiety. As always, the observations described here should be considered tentative pending replication. This is perhaps particularly true given the number and complexity of analyses we have performed.

## Conclusions

In sum, we found that higher levels of social anxiety were associated with increased activity in default mode network areas during anticipation and receipt of reward. Our findings and those of others suggest that social anxiety may be characterized not by decreased approach-oriented, appetitive motivation so much as by increased self-focused vigilance in the presence of potential rewards. Importantly, the MID task is not an overtly social task - potential gains and losses are of money only. These results suggest that social anxiety, even at sub-clinical levels, may affect reward processing more generally, not just processing explicitly related to social situations. Yet, it is worth noting that any task performed in an experimental setting is implicitly social - the presence of the experimenter and fMRI technician constitutes a social, or at least not isolated, situation. In other words, although the MID task does not involve social interaction, it does involve an evaluative context. Thus, the altered reward processing seen in socially anxious individuals may be particularly evident in performance or self-evaluative contexts, such as the MID task employed in our study, even in the absence of overt social interaction or cues.

## Abbreviations

ACC: Anterior cingulate cortex; BET: Brain Extraction Tool; BOLD: Blood oxygen level-dependent; DMN: Default mode network; FEAT: fMRI Expert Analysis Tool; FLIRT: FMRIB Linear Image Registration Tool; fMRI: Functional magnetic resonance imaging; FNE: Fear of Negative Evaluation subscale; FSL: FMRIB Software Library; MID: Monetary incentive delay; MNI: Montreal Neurological Institute; MPFC: Medial prefrontal cortex; PCC: Posterior cingulate cortex; RT: Reaction time; SAD-General: Social Avoidance and Distress in General Situations subscale; SAD-New: Social Avoidance and Distress in New Situations subscale; SAS-A: Social Anxiety Scale for Adolescents; SMC: Supplementary motor cortex; STAI: State-Trait Anxiety Inventory.

## Competing interests

The authors declare no competing interests.

## Authors’ contributions

ELM participated in study conception, contributed to data collection, conducted the data analysis, and took the lead on writing the manuscript. JPA helped conceive of the study design and coordination, aided in data collection, and contributed to writing the manuscript. JAC helped conceive of the study design and coordination, provided financial support, assisted in data interpretation, and contributed to writing the manuscript. All authors read and approved the final manuscript.

## Supplementary Material

Additional file 1 Supplementary analyses for anticipation and outcome phases. Click here for file

Additional file 2: Table S1 Main effects of gain > neutral and neutral > gain anticipation contrasts using whole brain analysis. **Table S2.** Negative associations with social anxiety in gain large > gain small anticipation contrast using whole brain analysis. **Table S3.** Positive associations with FNE and SAD-New subscales in gain > neutral anticipation contrast using whole brain analysis. **Table S4.** Main effects of loss > neutral and neutral > loss anticipation contrasts using whole brain analysis. **Table S5.** Main effects of gain > neutral and neutral > gain outcome contrasts using whole brain analysis. **Table S6.** Positive associations with social anxiety in gain large > gain small outcome contrast using whole brain analysis. **Table S7.** Positive associations with FNE and SAD-New subscales in gain > neutral outcome contrast using whole brain analysis. **Table S8.** Main effects of loss > neutral and neutral > loss outcome contrasts using whole brain analysis. **Table S9.** Positive associations with FNE and SAD-General subscales in loss > neutral outcome contrast using whole brain analysis. **Table S10.** Positive associations with social anxiety in gain > loss outcome contrast using whole brain analysis. Click here for file
