# Supplementary material for: Increased default mode network activity in socially anxious individuals during reward processing
Source: Biol Mood Anxiety Disord. 2014 Jul 23;4:7. doi: 10.1186/2045-5380-4-7 (PMC4114426; doi:10.1186/2045-5380-4-7)
Supplement: Additional file 1 — Supplementary analyses for anticipation and outcome phases. [file 2045-5380-4-7-S1.doc]

Supplementary Materials

**ANTICIPATION PHASE**

**Neutral *vs.* gain anticipation**

To investigate areas of decreased activity during reward anticipation, we also examined the reverse contrast (neutral cues contrasted with gain cues). This yielded predictable activations in areas related to the default mode network (DMN) , including bilateral middle frontal gyrus, superior frontal gyrus, precuneus, posterior cingulate cortex (PCC), superior temporal gyrus, middle temporal gyrus, and lateral occipital cortex, and left insula, orbitofrontal cortex, and inferior frontal gyrus (Table S1).

**Social anxiety and gain anticipation**

***Gain magnitude.*** To examine whether social anxiety was associated with differential neural activity across reward magnitudes, we compared anticipation of large rewards to anticipation of small rewards. This revealed that individuals higher in social anxiety showed decreased activity when anticipating large compared to small rewards in a cluster that peaked in the paracingulate and extended to the medial prefrontal cortex and frontal pole (Table S2).

***Social anxiety subscales***. To explore what facets of social anxiety might relate to altered neural processing during anticipation of gain, we examined each of the three subscales of the SAS-A separately. Both the FNE and SAD-New subscale scores corresponded with activity in a cluster overlapping with activity seen when using total social anxiety scores (Table S3). The cluster related to FNE peaked in the right intra-parietal sulcus and extended to the precuneus, PCC, right angular gyrus, and left lateral occipital cortex; the cluster related to SAD-New peaked in the precuneus and extended to the PCC. The SAD-General subscale showed no association with brain activity.

**Trait anxiety**

To explore overlap between social anxiety and trait anxiety, we used a mask created from regions related to social anxiety, thresholded at an uncorrected *P* value of *P* <0.005, and entered trait anxiety scores as a covariate. Using this method, trait anxiety corresponded with activity in small (10 to 20 voxels) clusters located in the left superior parietal lobule and precuneus.

**Neutral *vs.* loss anticipation**

Neutral minus loss anticipation cues yielded activity in areas including bilateral frontal pole, superior frontal gyrus, ACC, PCC, paracingulate, precuneus, intracalcarine cortex, and left frontal medial cortex, angular gyrus, supramarginal gyrus, lateral occipital cortex, and insula (Table S4).

**Social anxiety and loss anticipation**

***Loss magnitude.*** Comparing brain activity during anticipation of large losses with anticipation of small losses further revealed no relationship with social anxiety.

***Social anxiety subscales.*** Examining subscale scores (FNE, SAD-New, and SAD-General) revealed no association with brain activity during loss anticipation.

**OUTCOME PHASE**

**Neutral *vs.* gain outcomes**

To investigate areas of decreased activity during reward outcome cues, we examined the reverse contrast (neutral outcome cues contrasted with gain outcome cues). This yielded activations in areas including bilateral supplementary motor cortex, precentral gyrus, postcentral, gyrus, supramarginal gyrus, precuneus, PCC, parietal operculum cortex, Heschl’s gyrus, planum temporale, lateral occipital cortex, thalamus, brain stem, and left middle frontal gyrus and hippocampus (Table S5).

**Social anxiety and gain outcomes**

***Gain magnitude.*** Contrasting large gain outcome with small gain outcome yielded three clusters of activity positively related to social anxiety (Table S6). The first cluster peaked in the left lingual gyrus and extended to the left intracalcarine cortex, fusiform gyrus, and lateral occipital cortex. The second cluster peaked in the right intracalcarine cortex and extended to the left lateral occipital cortex, occipital pole. The third cluster peaked in the right superior parietal lobule and extended to the right postcentral gyrus, lateral occipital cortex, and supramarginal gyrus.

***Social anxiety subscales***. We next examined how the SAS-A subscales might relate to altered neural processing during gain outcomes. As with gain anticipation, both the FNE and SAD-New subscale scores corresponded with activity during gain outcomes in regions similar to those seen when using total social anxiety scores, whereas the SAD-General subscale showed no relationship with brain activity (Table S7). The FNE subscale associated with activity in three clusters. The first peaked in the precuneus and extended to left precentral and postcentral gyrus, bilateral superior parietal lobule and supramarginal gyrus; the second peaked in the left lateral occipital cortex and extended to the left lingual gyrus; the third peaked in the right middle frontal gyrus and extended to the right superior frontal gyrus and precentral gyrus. The SAD-New subscale associated with activity in one cluster that peaked in the left superior parietal lobule and extended to the right superior parietal lobule, bilateral supramarginal gyrus, lateral occipital cortex, precuneus, and left postcentral gyrus.

**Trait anxiety**

Using a mask created from regions related to social anxiety and thresholded at an uncorrected *P* value of *P* <0.005, we entered trait anxiety scores as a covariate. Using this method, trait anxiety corresponded with activity in small (10 to 20 voxels) clusters located in bilateral superior parietal lobules.

**Neutral *vs.* loss outcomes**

The reverse contrast (neutral outcome cues contrasted with loss outcome cues) yielded activations in the precentral gyrus, postcentral gyrus, supplementary motor cortex, and PCC (Table S8).

**Social anxiety and loss outcomes**

***Loss magnitude.*** Comparing large loss outcomes to small loss outcomes did not yield any relationship with social anxiety.

***Social anxiety subscales.*** Examining subscale scores of the SAS-A did reveal facets of social anxiety related to neural activity during loss outcomes (Table S9). Higher scores on the FNE subscale corresponded with increased activity in a cluster including the supplementary motor cortex, precentral gyrus, superior frontal gyrus, and paracingulate. Conversely, higher scores on the SAD-General subscale corresponded with decreased activity in two clusters, the first peaking in the PCC and extending to the precuneus, and the second peaking in the left lateral occipital cortex. The SAD-New subscale showed no relationship with brain activity.
